# Decoding the impact of exercise and αCGRP signaling on murine post-traumatic osteoarthritis progression

**DOI:** 10.1186/s13075-025-03589-6

**Published:** 2025-06-21

**Authors:** Patrick Pann, Paul Kalke, Verena Maier, Nicole Schäfer, Hauke Clausen-Schaumann, Arndt F. Schilling, Susanne Grässel

**Affiliations:** 1 Dept. of Orthopaedic Surgery, Experimental Orthopaedics, Center for Medical Biotechnology, University of Regensburg, ZMB im Biopark 1 Am Biopark 9, 93053 Regensburg, Germany; 2 Department of Trauma Surgery, Orthopedics and Plastic Surgery, University Medicine Göttingen, Göttingen, Germany; 3 Center for Applied Tissue Engineering and Regenerative Medicine (CANTER), University of Applied Sciences Munich, Munich, Germany

**Keywords:** Osteoarthritis, Destabilization of the medial meniscus, Alpha-calcitonin gene-related peptide, Exercise, Bone, Cartilage

## Abstract

**Background:**

Osteoarthritis (OA) is a chronic degenerative joint disease characterized by cartilage breakdown, subchondral bone remodeling, and inflammation. Mechanical stress, such as exercise, can influence OA progression, acting as either a therapeutic intervention or a risk factor depending on intensity. The sensory neuropeptide αCGRP plays a role in modulating cartilage, bone, and inflammatory responses, making it a potential mediator of exercise effects on OA. This study investigated the impact of αCGRP deficiency and exercise intensity on OA progression in a post-traumatic murine model.

**Methods:**

OA was induced in male αCGRP knockout (KO) and wild type (C57Bl/6J) mice via destabilization of the medial meniscus (DMM). Mice underwent moderate or intense treadmill exercise for up to 6 weeks (8 weeks post-surgery). Histological analyses were performed to assess cartilage degradation. Subchondral and metaphyseal bone morphology as well as cartilage stiffness were evaluated by nanoCT and atomic force microscopy (AFM), respectively. Serum inflammatory markers were analyzed using multiplex immunoassays.

**Results:**

Serum levels of proinflammatory markers were elevated in αCGRP-deficient mice, particularly after intense exercise, independent of OA progression. DMM surgery induced significant cartilage degradation. Gross cartilage morphology was not influenced by exercise intensity or αCGRP deficiency, but αCGRP deficiency prevented articular cartilage extracellular matrix stiffening after DMM and intense exercise. Subchondral bone sclerosis was induced by αCGRP deficiency and DMM but mitigated by intense exercise. In metaphyseal bone, intense exercise induced trabecular loss in αCGRP-deficient mice.

**Conclusions:**

This study highlights αCGRP as an intrinsic regulator of joint and bone responses to mechanical loading during OA. While cartilage degradation after DMM and treadmill exercise was unaffected by lack of αCGRP, its deficiency altered ECM stiffness, bone remodeling, and inflammatory responses. These findings position αCGRP as a critical regulator of joint homeostasis, particularly for bone health during running exercise and OA progression.

**Supplementary Information:**

The online version contains supplementary material available at 10.1186/s13075-025-03589-6.

## Introduction

Osteoarthritis (OA) is a chronic and degenerative joint disease affecting millions of people worldwide, particularly adults over the age of 40 [1]. It is characterized by a progressive breakdown of articular cartilage, changes in subchondral bone structure, synovial inflammation, and osteophyte formation, which results in pain, stiffness, and reduced joint mobility [2, 3]. Its multifactorial etiology includes high age, genetics, obesity, trauma, and mechanical stress [4]. OA poses a significant socioeconomic burden, contributing to rising healthcare costs, loss of productivity, and reduced quality of life [5]. Despite scientific advances, OA remains incurable, with existing treatments mostly focusing on symptom relief rather than disease modification [2].

Mechanical stress i.e. exercise plays a dual role in OA pathophysiology, acting as both a risk factor and a therapeutic intervention. High-impact or repetitive loading, particularly following joint injury, increases the risk of post-traumatic OA (PTOA) due to mechanical damage and impaired joint homeostasis [6]. Conversely, appropriately prescribed exercise therapy is one of the most effective non-pharmacological treatments for OA. Aerobic, resistance, and neuromuscular exercises improve pain, muscle strength, and functional outcome. Systematic reviews report that both land-based and aquatic exercise programs significantly reduce OA symptoms without worsening disease progression [4]. However, the precise mechanisms through which exercise influences OA development and progression remain incompletely understood.

The sensory nervous system plays a pivotal role in joint homeostasis and OA pathogenesis. Sensory neuropeptides such as substance P and alpha-calcitonin gene-related peptide (αCGRP) are released by nerve endings innervating joint tissues, including meniscus, subchondral bone, and synovium [7]. These neuropeptides modulate pain perception, inflammation, and tissue repair, linking the nervous system to joint pathology [8, 9]. αCGRP exerts trophic effects on chondrocytes and osteoblasts, promoting bone formation and cartilage maintenance. However, αCGRP can also contribute to OA pathology by stimulating osteophyte formation, synovitis, and pain [2]. Furthermore, murine models of OA have shown that αCGRP deficiency accelerates cartilage degeneration and alters subchondral bone structure, highlighting its protective and pathological roles [3]. Given that exercise influences neuropeptide release and joint mechanics, αCGRP may be a key mediator of exercise-induced effects on OA progression. We therefore hypothesize that αCGRP participates in the interplay of OA pathogenesis and exercise. Therefore, this study aims to elucidate the effect of αCGRP deficiency in a surgically induced OA mouse model combined with forced treadmill exercise, providing novel insights into neuropeptide-mediated mechanisms of joint degeneration.

## Materials and methods

### Animals

The origins and housing conditions of the experimental animals were identical to those in our previous study by Muschter et al. 2020 [3]. Male C57Bl/6J wild-type (WT) mice, aged 8–10 weeks, were obtained from Charles River Laboratories (Sulzfeld, Germany) and acclimated to standard housing conditions with a 12-hour dark/light cycle until they reached 12 weeks of age. Age- and sex-matched αCGRP−/− global knockout mice generated by R.B. Emeson [10] were a kind gift from T. Schinke and M. Amling (Eppendorf University Hospital Hamburg) and were bred back on a C57Bl/6J background. C57Bl/6J (WT) mice were included in the study for comparative analysis. The animals had *ad libitum* access to food and water. In total 102 WT and 107 KO animals were used in this study. All experiments were approved by the local ethics committee (Regierung von Unterfranken, Bavaria, RUF-55.2.2-2532-2-1253, approval date: November 5, 2020).

### Destabilization of the medial meniscus (DMM)

OA was induced by surgical destabilization of the medial meniscus (DMM) as described by Glasson [11]. Since the DMM surgical model is sex hormone-dependent, only male mice were used [12]. Briefly, after intraperitoneal anesthesia with fentanyl (0.05 mg/kg), medetomidine (0.5 mg/kg), and midazolam (5 mg/kg), a 3 mm skin incision was made between the distal patella and proximal tibial plateau of the right leg to expose the knee joint. A 1–2 mm incision medial to the patellar tendon opened the joint capsule. The medial meniscotibial ligament was carefully dissected using micro scissors to induce OA. Sham surgery was performed in a separate group of animals by visualizing the ligament without dissection. The skin was sutured, and the anesthesia was antagonized using Atipamezol (2.5 mg/kg) and Flumazenil (0.5 mg/kg). The animals received analgesia (0.1 mg/kg buprenorphine) immediately after surgery and after 8, 16, and 24 h. Postoperatively, animals were allowed free movement for recovery.

### Treadmill exercise

One week after surgery, mice underwent a one-week habituation period on a motorized treadmill (Ugo Basile, Gemonio, Italy). Forced exercise protocols began two weeks post-surgery, with animals randomly assigned to one of two exercise intensity groups: moderate (10 m/min, 5° incline) or intense (16 m/min, 15° incline). Mice exercised for 30 min per day, five days per week, for either two or six weeks. Exercise parameters were selected based on published protocols reporting either reduced (moderate) or accelerated (intense) OA progression [13, 14]. The moderate speed and incline were chosen to enable a fast-paced walk without inducing running or jumping, while maintaining high motivation and compliance in the animals. The intense protocol represented the maximum speed that animals could sustain over the full exercise duration, with the steeper incline designed to increase mechanical load on the hind limbs. At four and eight weeks post-surgery, animals were killed using carbon dioxide. Immediately after euthanasia, blood was collected via intracardiac puncture. Knee joints were collected and processed directly for histological analysis. For atomic force microscopy (AFM) and nano-computer tomography (CT), animals were killed eight weeks after surgery and the samples were prepared as described in the respective methods.

### Serum analysis

Blood samples were drawn via intracardiac puncture eight weeks post-surgery after euthanasia and serum was collected after centrifugation. Multiplex immunoassays were performed to measure the expression of proinflammatory and OA-associated factors. Serum samples were analyzed using a Bio-Plex 200 Multiplex Reader (Bio-Rad Laboratories GmbH, Feldkirchen, Germany) with a Mouse ProcartaPlex Mix&Match 14-plex assay (PPX-14-MX324DH, Thermo Fisher Scientific, Vienna, Austria) for measuring the factors Eotaxin (CCL11), GRO alpha (CXCL1), IFN-γ, IL-1β, IL-6, IP-10 (CXCL10), Leptin, MCP-1 (CCL2), MIG (CXCL9), MIP-1α (CCL3), MIP-1β (CCL4), RANKL, RANTES (CCL5), and VEGF-A.

### Histology and OA scoring

Histological sample preparation and OA scoring were performed as described in our previous study [3]. Immediately after dissection, knee joint samples were fixed in 4% paraformaldehyde/ PBS for 24 h and decalcified in 20% EDTA in ddH_2_O, pH 7.4 for 8 weeks. Decalcification was carried out without shaking and ETDA replacement twice weekly. Samples were embedded in paraffin and 6 μm frontal sections were taken using a microtome. For evaluation of cartilage degradation, 6 adjacent section pairs in 60–90 μm intervals (12 sections total) were deparaffinized, rehydrated and stained with Safranin O, Weigerts iron hematoxylin and Fast Green and scored by two independent, blinded investigators according to the modified OARSI guidelines described in Table 1 [15]. Sections were scanned with 10× magnification using the BZ-X810 digital microscope (KEYENCE Deutschland GmbH, Neu-Isenburg, Germany). Maximum OARSI scores of the lateral and the medial femur condyle and tibia plateau were averaged, respectively.

**Table 1:** OARSI guidelines for assessment of murine cartilage degradation (modified after [15])

Grade	Osteoarthritic damage
0	No damage
0.5	Loss of Safranin O staining without structural changes
1	Small fibrillations without loss of cartilage/uneven surface
2	Vertical clefts and erosions down to the layer immediately below the superficial layer and some loss of surface lamina
3	Vertical clefts/erosions to the calcified cartilage < 25% of the articular surface
4	Vertical clefts/erosions to the calcified cartilage 25–50% of the articular surface
5	Vertical clefts/erosions to the calcified cartilage 50–75% of the articular surface
6	Vertical clefts/erosions to the calcified cartilage > 75% of the articular surface

### Indentation-type atomic force microscopy

Atomic force microscopy (AFM) analysis was adapted from the protocol described in our previous study [3], with the use of a newer-generation AFM and microscopy system enabling larger-area mapping and higher vertical tip velocities. AFM was solely used in the quantitative imaging mode (QI™). For QI™, native, non-fixated knee tissues 8 weeks after DMM or Sham surgery were-snap frozen and cut in frontal direction into 20 μm slides using a cryotome (Leica CM 1950). To maintain tissue integrity throughout AFM measurements, transparent adhesive tape (tesafilm Nr.: 57330-00000) was used to obtain the tissue sections which were then attached to a glass slide via a double adhesive tape (tesafilm Nr.: 56661-00002). QITM was conducted using a NanoWizard IV AFM (Bruker Nano GmbH, Berlin, Germany) in combination with an inverted optical microscope (Stellaris 8, Leica Microsystems GmbH, Wetzlar, Germany) for precise lateral positioning of the AFM-tip. The influence of external noise was reduced by placing the whole set-up on an active vibration isolation table (Accurion, Park Systems Corporation, Korea) inside a soundproof box. Indentation experiments were performed with silicone-nitride cantilevers (MLCT, Cantilever F, Bruker, Berlin, Germany) with a nominal spring constant of 0.6 N/m, a nominal tip radius of 20 nm and a pyramidal tip shape. For each cantilever the spring constant was determined individually using the thermal noise method [16]. During QITM, the tissue sections were immersed in PBS (Bio&Sell (D)PBS, without Ca2 + and Mg2+, Feucht, Germany). The frontal cut of the knee tissues allowed a clear identification of the medial tibia plateau and its individual cartilage zones (superficial, middle and deep zone). In all three zones the extracellular matrix (ECM) of the cartilage was investigated by QITM force maps using the frontally cut sections right after defrosting them at room temperature. Each force map held 128 × 128 force-indentation curves equally distributed over an area of 15 × 15 μm². The vertical tip velocity was 300 μm/s throughout all QITM measurements. In total, three force maps for each cartilage zone, surgery type, genotype, and type of exercise were assessed by using three different sections per animal. This was followed by a three-step analysis of the recorded data. First, the Young’s Modulus was extracted by fitting the Hertz model modified for a pyramidal indenter to the approach part of the force-indentation curves up to an indentation depth of 1 μm using the JPK Data Processing Software. The remaining steps were conducted using the software CANTER Processing Toolbox (https://github.com/CANTERhm/CANTER_Processing_Tool). In the second step, error-prone force curves (e.g. artifact regions, R² < 0.975, cell regions) were sorted out to ensure a conclusive ECM-specific result. Third, stiffness distributions were generated, and the two maxima of the bimodal distributions were determined by fitting a linear combination of two Gaussian distributions to the data [17]. Loparic et al. observed a similar bimodal nano-stiffness in mature articular cartilage. This group demonstrated that the first peak can be attributed to the proteoglycan phase and the second peak to the collagen fibrils [18].

### NanoCT analysis

NanoCT analysis was adapted from the protocol described in our previous study [3]. Immediately after dissection knee joint samples were fixed in 4% paraformaldehyde/ PBS for 24 h and stored at 4 °C in 70% ethanol. Nano-CT analysis was performed as described by Muschter et al. [3]. For imaging, knee joints were scanned using a Scanco µCT 50 device (Scanco Medical, Brüttisellen, Switzerland) under air conditions. The imaging parameters included a source voltage of 90 kVp and a current of 88 µA. To reduce beam hardening effects, a 0.50 mm aluminum filter was employed. All scans were performed with an isotropic voxel size of 6.8 μm and an integration time of 1000 ms, aimed at generating 3D overviews of the femorotibial joint, including the assessment of topographical alterations in the metaphyseal region, meniscal ossicles, and potential osteophyte formation. Image reconstruction was conducted using Scanco’s OpenVMS software. Bone morphometry indices were determined within two defined volumes of interest (VOIs). The first VOI encompassed the metaphyseal region, specifically a 1.2 mm³ area located 300 mm distal to the epiphyseal line, for evaluating sub-articular trabecular morphometry. The second VOI made up an approximately 0.2 mm³ area within the medial epiphysis, positioned between the lower margin of the subchondral bone plate and the epiphyseal line. In both VOIs, threshold values optimized for Scanco’s OpenVMS software were applied (lower threshold: 685.3 mg HA/cm³, upper threshold: 3000 mg HA/cm³, Gauss Sigma: 0.8, Gauss Support: 1). Manual contouring was performed, excluding the endocortical surface, in alignment with standard guidelines. Subchondral bone plate thickness was measured using ImageJ software in three equidistant coronal cross-sections of the joint. The results for each condyle were reported as the mean ± SEM of 60 spots across the three coronal planes. To assess tibial plateau morphology, the lengths of the lateral and medial condyles were measured. These were defined as the distances from the condylar center near the trochlear groove to the lateral or medial prominence. Heterotopic ossification of the meniscus was evaluated by manually delineating and segmenting anterior meniscal ossicles. The VOIs for Sham and DMM mice were approximately 0.4 mm³ and 1.0 mm³, respectively, accounting for the irregular surface expansion of ossicles due to post-traumatic alterations. Quantitative parameters included bone volume (BV), bone mineral density (BMD), bone surface (BS), and the BS/BV ratio for the anterior meniscus, with comparisons performed across the study groups.

### Statistical analysis

For biometric planning, the program GPower 3.1.9 (Heinrich Heine University, Düsseldorf) and data from a previous study by our research group involving wild-type and αCGRP-deficient mice were used [3]. Group size estimation for multiple group comparisons was performed using an F-test (ANOVA: fixed effects, omnibus, one-way) with an assumed power of 80% and a type I error probability of 0.05, applying Bonferroni correction for multiple comparisons.

GraphPad Prism Software 8.0.2 (San Diego, CA, USA) was used for statistical analysis and graph preparation. Outliers were removed from data using GraphPad Prisms ROUT method with Q = 1% for OARSI scores and Q = 5% for serum concentrations [19]. For the analysis of OARSI scores groups were compared for genotype-, surgery- and exercise-dependent differences using Kruskal-Wallis with Dunn’s post-hoc test for multiple comparisons. Serum concentrations as well as NanoCT and AFM data were compared for genotype-, surgery, and exercise-dependent differences using Two-Way ANOVA with Tukey’s multiple comparisons test. OARSI scores and serum concentrations are depicted as box plots showing the median and the upper and lower interquartile range with whiskers encompassing minimum and maximum values. NanoCT and AFM results are depicted as bar plots showing the mean and the standard deviation in error bars. Exemplary detailed AFM results are also provided as histograms.

## Results

### DMM induces medial cartilage degradation

**Fig. 1:**
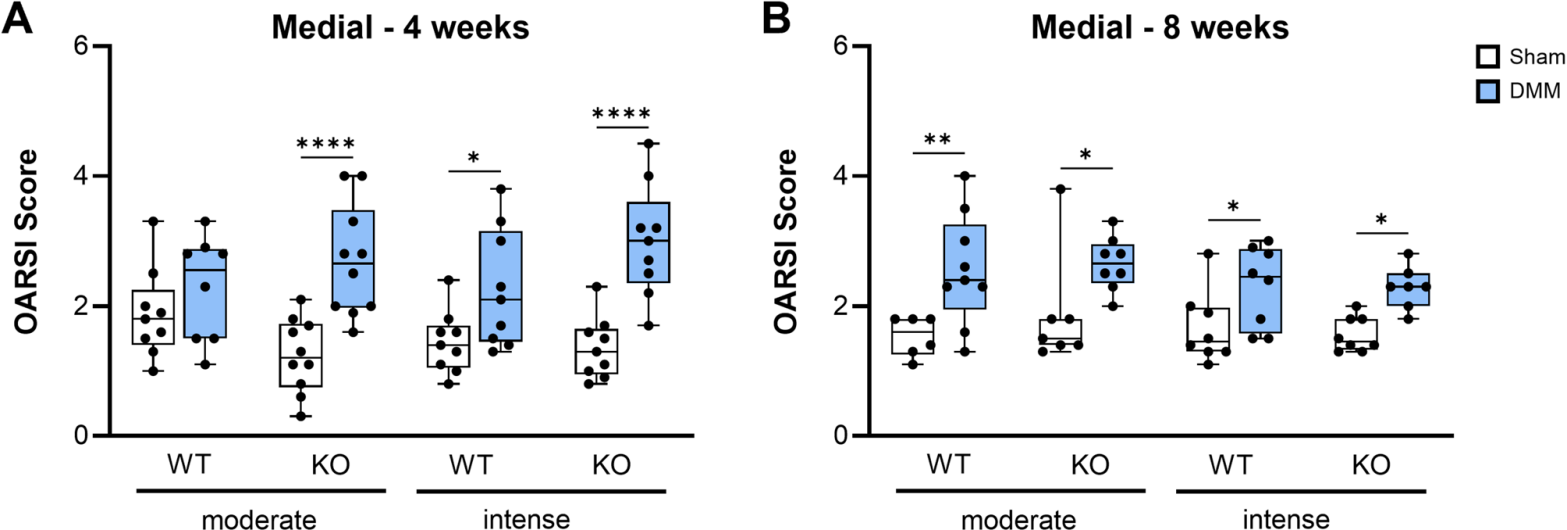
Impact of αCGRP deficiency and exercise intensity on medial cartilage degradation after OA induction Cartilage was evaluated for grades of destruction according to the OARSI guidelines for murine OA. Cartilage of the right knee joints of WT and KO mice exposed to moderate or intense exercise were graded 4 weeks (**A**) and 8 weeks (**B**) after Sham or DMM surgery. Means of the maximal OARSI scores of the medial tibial and femoral cartilage together were compared. Statistical analysis is using Kruskal-Wallis and Dunn’s test for multiple comparisons. * *p* < 0.05, ** *p* < 0.01. *N* = 6–10

Articular cartilage matrix degradation was assessed in knee joint sections stained with Safranin O at 4 and 8 weeks after surgery, corresponding to 2 and 6 weeks of treadmill exercise. After Sham surgery, mice of all groups exhibited OARSI scores between 1 and 2 in lateral and medial cartilage – combined femoral and tibial scores - compartments. DMM surgery significantly increased medial cartilage degradation in comparison to Sham mice at both time points (Fig. 1A, B; Suppl. Figure 1), while almost no changes could be observed in the lateral knee region (Suppl. Figure 1, 2). Across all groups, the knockout of αCGRP (αCGRP-/-), the exercise intensity, and the sampling time point did not affect OARSI scores.

### αCGRP deficiency prevents cartilage stiffening after OA induction and exercise

**Fig. 2:**
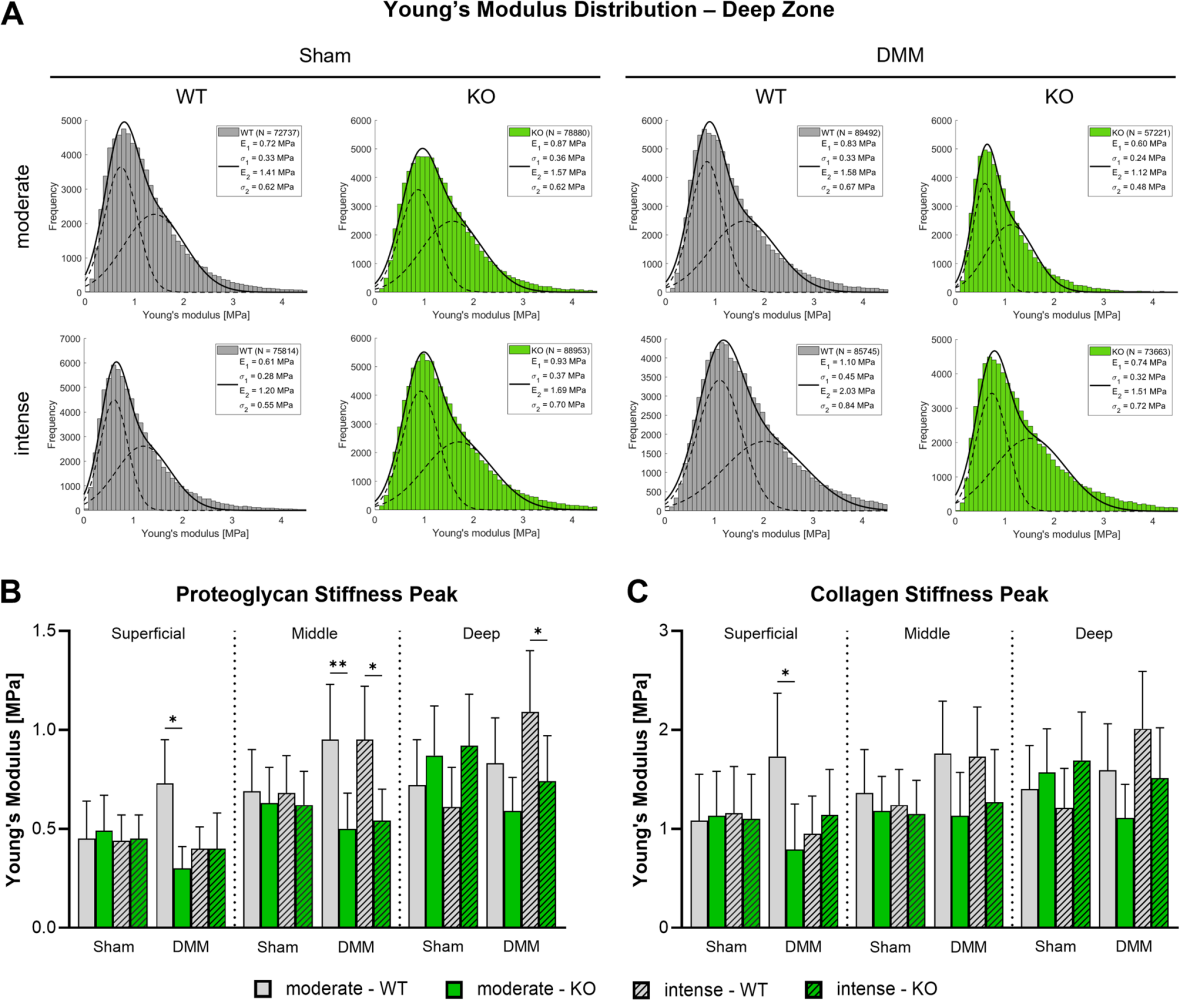
Atomic force microscopy-based analysis of cartilage matrix stiffness in αCGRP deficient mice after OA-induction and forced exercise Analysis of articular cartilage of the right knee joint of WT and KO mice exposed to moderate and intense exercise at 8 weeks after DMM or Sham surgery **A**) Histograms of Young’s modulus (stiffness) distribution of the deep zone cartilage matrix (histograms of superficial and middle zone are in the supplementary material). The black line in each histogram represents a fit to the data using a linear combination of two Gaussian distributions. The dashed black lines show the individual Gaussian distributions representing the proteoglycan (E1, σ1) and the collagen (E2, σ2) Young’s moduli, respectively, as described in detail in the methods section. *N* = 3 **B-C**) Mean Young’s modulus (stiffness) of the proteoglycan (**B**) and collagen (**C**) peaks of superficial zone, middle zone, and deep zone cartilage. Bars show mean ± standard deviation. Statistical analysis using Two-Way ANOVA and Tukey’s multiple comparisons test. * *p* < 0.05, ** *p* < 0.01. *N* = 3

Stiffness of the superficial, middle and deep articular cartilage matrix zones evaluated by proteoglycan and collagen content was analyzed 8 weeks after surgery using AFM. While no differences across all groups were found in Sham mice, proteoglycan associated stiffness peaks were significantly influenced by exercise intensity and the knockout of αCGRP after DMM surgery (Fig. 2A, B; Suppl. Figure 3, 4). Specifically, in the superficial zone a significant increase in stiffness was found in WT mice after moderate exercise in comparison to KO mice. In contrast, deep zone proteoglycan stiffness was only increased in WT mice after intense exercise. In the middle zone, an increase compared to KO mice was observed after both moderate and intense exercise in WT mice. Notably, the stiffness of the proteoglycan matrix in αCGRP-deficient KO mice remained unaffected (no significant changes) after OA induction and intense exercise. The stiffness of the collagen-associated matrix was generally about twice as high as the proteoglycan associated stiffness but showed only comparable differences between the groups by trend except for the superficial zone with a significant increase after moderate exercise in WT (Fig. 2A, C; Suppl. Figure 3, 4).

### αCGRP deficiency and intense exercise attenuate meniscal ossicle formation after OA-induction

**Fig. 3:**
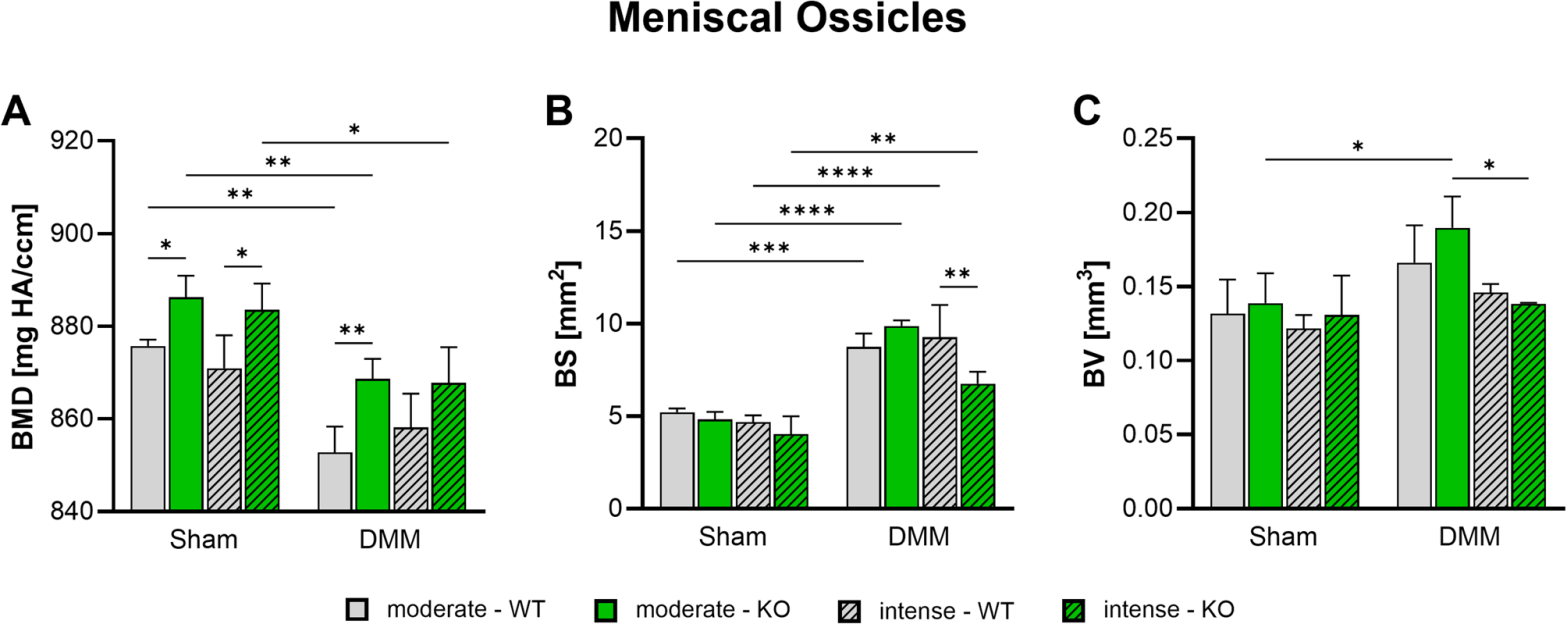
Effect of αCGRP deficiency and forced exercise on meniscal ossification after OA-induction Ultra-high resolution nanoCT analysis of medial and lateral meniscal ossicle formation in WT and KO mice exposed to moderate or intense exercise at 8 weeks after DMM or Sham surgery. Analysis of (**A**) bone mineral density (BMD), (**B**) bone surface (BS), and (**C**) bone volume (BV). Statistical analysis using Two-Way ANOVA and Tukey’s multiple comparisons test. * *p* < 0.05, ** *p* < 0.01, *** *p* < 0.001, **** *p* < 0.0001. *N* = 3

Ectopic meniscal bone formation was measured 8 weeks after surgery via nanoCT analysis. OA-induction by DMM significantly decreased bone mineral density (Fig. 3A) while increasing bone surface area (Fig. 3B) in all groups. Bone volume was only increased in αCGRP KO mice after moderate exercise (Fig. 3C). αCGRP deficiency on the other hand significantly increased mineral density irrespective of surgery or exercise intensity (Fig. 3A). Additionally, bone surface area was found to be reduced in αCGRP-/- mice after intense exercise (Fig. 3B). The OA-induced increase in bone volume in WT and αCGRP-/- mice returned to Sham levels after intense exercise (Fig. 3C).

### DMM induces medial osteophyte formation

**Fig. 4:**
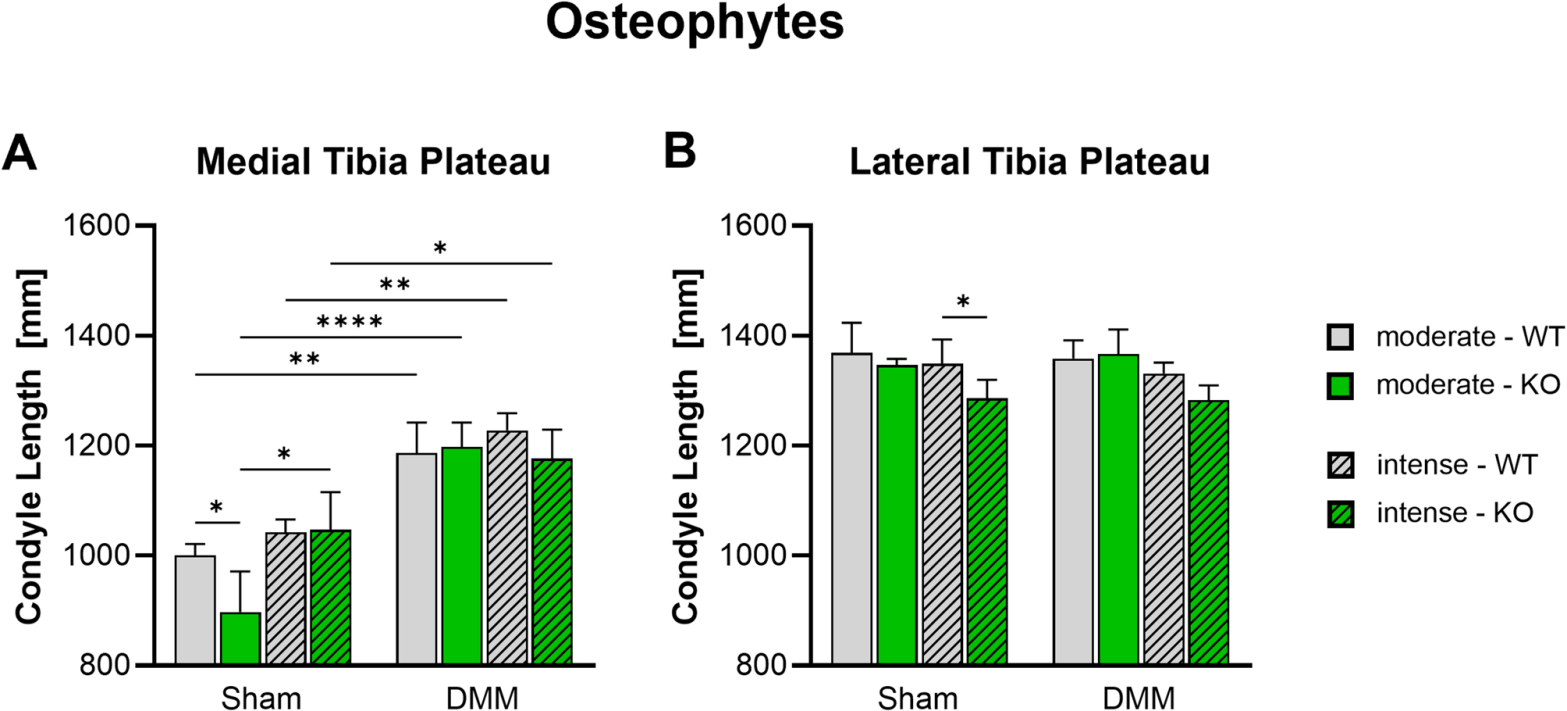
Effect of αCGRP deficiency and forced exercise on osteophyte formation after OA-induction Ultra-high resolution nanoCT analysis of the medial (**A**) and lateral (**B**) tibia plateau diameter in WT and KO mice exposed to moderate or intense exercise at 8 weeks after DMM or Sham surgery. Statistical analysis using Two-Way ANOVA and Tukey’s multiple comparisons test. * *p* < 0.05, ** *p* < 0.01, *** *p* < 0.001, **** *p* < 0.0001. *N* = 3

Medial and lateral tibial diameter was measured 8 weeks after surgery via nanoCT analysis to assess osteophyte formation. DMM surgery induced significant osteophyte formation at the medial tibia plateau of all groups. Furthermore, αCGRP deficient Sham mice showed a significantly reduced medial tibia diameter after moderate exercise. A significant effect that was not observed in DMM mice (Fig. 4A). At the lateral plateau side no differences were seen except for a reduced diameter in αCGRP-/- mice after Sham surgery and intense exercise. Again, this effect could not be observed in DMM mice (Fig. 4B).

### Medial subchondral bone sclerosis is induced by αCGRP deficiency and OA but mitigated by intense exercise

**Fig. 5:**
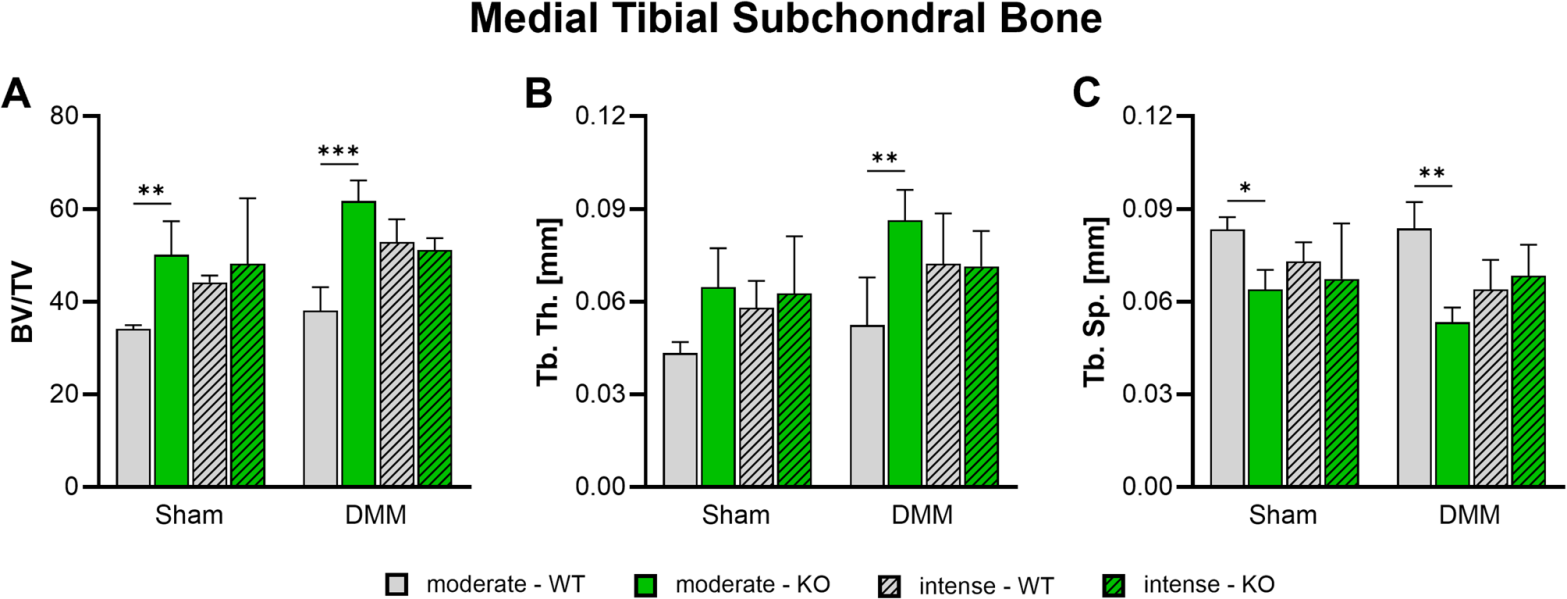
Effect of αCGRP deficiency and forced exercise on subchondral bone morphology after OA-induction Ultra-high resolution nanoCT analysis of the subchondral bone of the medial tibia in WT and KO mice exposed to moderate or intense exercise at 8 weeks after DMM or Sham surgery. Analysis of (**A**) bone volume to total volume ratio (BV/TV), (**B**) trabecular thickness (Tb. Th.), (**C**) trabecular separation (Tb. Sp.). Statistical analysis using Two-Way ANOVA and Tukey’s multiple comparisons test. * *p* < 0.05, ** *p* < 0.01, *** *p* < 0.001. *N* = 3

Changes in medial subchondral bone morphology were measured 8 weeks after surgery via nanoCT analysis. Here, αCGRP deficiency led to a significant induction of bone sclerosis after moderate exercise correlated to an increase in bone volume/total volume ratio independent of surgery type (Fig. 5A). This was accompanied by an increase in trabecular thickness (Fig. 5B) and a decrease in trabecular separation (Fig. 5C) in comparison to WT mice. DMM surgery seemed to augment this effect, while it did not alter the bone morphology of WT animals. Like αCGRP deficiency, intense exercise by trend also led to sclerotic changes but at the same time mitigated the effects induced by the knockout of αCGRP. The number of trabeculae (Tb. N.) and the subchondral bone plate (SBP) thickness remained unchanged in all groups (Suppl. Figure 5).

### αCGRP deficiency affects medial metaphyseal trabecular bone structure after DMM and exercise

**Fig. 6:**
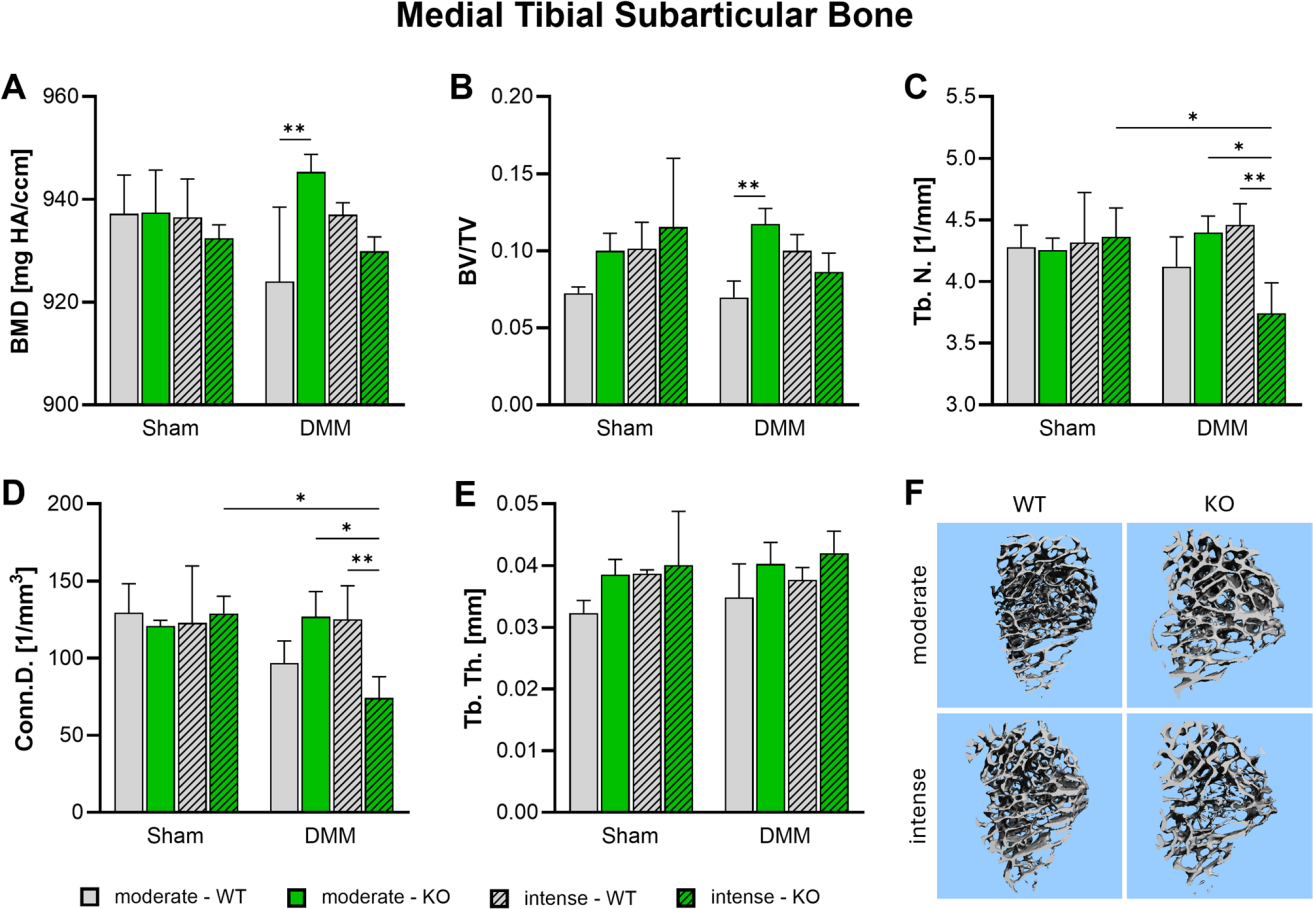
Effect of αCGRP deficiency and forced exercise on metaphyseal bone morphology after OA-induction Ultra-high resolution nanoCT analysis of the metaphyseal bone of the medial tibia in WT and KO mice exposed to moderate or intense exercise at 8 weeks after DMM or Sham surgery. Analysis of (**A**) bone mineral density (BMD), (**B**) bone volume to total volume ratio (BV/TV), (**C**) trabecular number (Tb. N.), (**D**) Connectivity Density (Conn. D.), and (**E**) trabecular thickness (Tb. Th.). (**F**) Representative images highlighting morphological differences in WT and KO mice after DMM and moderate or intense exercise. Statistical analysis using Two-Way ANOVA and Tukey’s multiple comparisons test. * *p* < 0.05, ** *p* < 0.01. *N* = 3

After the discovery of increased subchondral bone sclerosis, nanoCT analyses were extended to the underlying trabecular metaphyseal bone. Here, no significant differences were seen in Sham mice of all groups. However, after OA-induction αCGRP-/- mice showed a significantly higher BMD and BV/TV ratio compared to WT animals after moderate exercise (Fig. 6A, B, F). Intense exercise mitigated these effects. Notably, αCGRP deficiency led to a significant drop in trabecular number and connective density after DMM in combination with intense exercise (Fig. 6C, D, F). Trabecular thickness remained largely unaffected in all groups (Fig. 6E).

### αCGRP deficiency raises inflammatory serum markers, especially after DMM and intense exercise

**Fig. 7:**
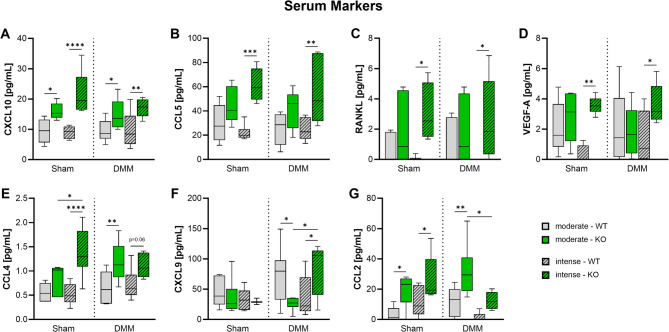
OA-associated serum factors in αCGRP deficient mice after OA-induction and forced exercise Serum marker concentration of WT and KO mice exposed to moderate or intense exercise at 8 weeks after Sham or DMM surgery. Statistical analysis using Two-Way ANOVA and Tukey’s multiple comparisons test. * *p* < 0.05, ** *p* < 0.01, *** *p* < 0.001, **** *p* < 0.0001. *N* = 6

Blood samples were taken 8 weeks after surgery and serum concentrations of proinflammatory and OA-associated markers were measured (Fig. 7). While αCGRP deficiency mostly led to a significant increase in serum levels of all quantifiable markers, for some serum factors this was only the case in combination with DMM surgery or intensive training.

CXCL10, CCL5, RANKL, and VEGF-A serum concentrations did not change after OA-induction in both WT and αCGRP-/- mice (Fig. 7A-D). Among these only CXCL10 exhibited significantly increased serum levels in all KO groups irrespective of surgery or exercise intensity (Fig. 7A). CCL5 (Fig. 7B) and RANKL (Fig. 7C) concentrations were significantly increased in KO mice after intense exercise but only by trend after moderate exercise. VEGF-A levels were solely increased after intense exercise (Fig. 7D).

In contrast, CCL4 levels in serum of Sham KO mice were only increased after intense exercise while DMM KO mice showed significant increases in both exercise regimen (Fig. 7E). Similarly, CXCL9 levels of KO mice were unaffected after Sham surgery but significantly increased after DMM surgery in combination with intense exercise (Fig. 7F). CCL2 levels exhibited a unique pattern, with all KO groups showing elevated levels compared to WT mice, except in DMM mice after intense exercise (Fig. 7G). Serum concentrations of IFN-γ, IL-1β, IL-6, Leptin, and CCL3 were below the detection limit of the multiplex instrument.

## Discussion

Mechanical stress as exercise is a double-edged sword in OA pathology. OA induction via DMM, ACL-T (anterior cruciate ligament transection) or high fat diet in rodent models have shown that moderate exercise prevents cartilage damage and delays osteosclerosis [13, 20–23]. Intense exercise after OA induction on the other hand is reported to be detrimental to the OA affected joint and to increase the severity of OA lesions [21, 24, 25]. Furthermore, treadmill exercise was shown to induce OA on its own without the need for destabilization surgery or diet [14, 26, 27]. Although the underlying molecular mechanisms of these outcomes are not yet fully understood, the sensory neuropeptide αCGRP might be a significant link to investigate as it was shown to modulate cartilage maintenance, bone formation, joint inflammation, gait and pain [2, 3, 8]. Therefore, this study investigated the effect of αCGRP deficiency and exercise intensity on OA progression.

As expected, we observed that DMM surgery reliably induced medial tibial and femoral cartilage degradation. However, no differences between WT and αCGRP-/- mice and between moderate and intense exercise regimen could be detected 4 and 8 weeks after DMM surgery. Instead, it seems that both exercise regimen increased medial and lateral cartilage degradation equally in all groups in comparison to previous studies without exercise [3]. Research groups reporting cartilage preservation after moderate exercise generally used no incline, putting less stress on the hind legs, lower treadmill speeds or provided a longer rest period between surgery and first exercise. In addition, Iijima et al. used rats, for which their protocols may have represented a lower stress level due to larger animal size [13, 21, 22]. Thus, our moderate protocol imposed a higher load, resulting in cartilage degeneration comparable to that observed with their intense exercise regimen in rats.

While there was no obvious macromolecular histomorphometric phenotype, genotype- and exercise-intensity-dependent changes in cartilage matrix structure were observed, reflected in proteoglycan and collagen stiffness alterations. While moderate exercise after OA induction increased proteoglycan and collagen stiffness in the superficial and middle zone, intense exercise induced stiffening in the middle and deep zone. Changes in the composition and structural alignment of extracellular matrix (ECM) components, such as large proteoglycans and fibrillar collagens, are associated with OA [28, 29]. During the onset of OA, the overall stiffness of cartilage measured at larger scales decreases, whereas the stiffness of finer structures, such as collagen fibrils, measured at the nanoscale, increases [30, 31]. αCGRP deficiency prevented the stiffening of proteoglycan and collagen fibers after OA induction, regardless of exercise intensity, suggesting a role in the rearrangement of cartilage ECM after mechanical stress. In comparison our previous study without forced exercise revealed cartilage stiffening in αCGRP-/- mice after OA-induction [3]. While the number of αCGRP-positive sensory nociceptive nerve fibers in the murine OA joint, in particular in the synovium, is assumed to be increased [32], data on the expression of the αCGRP receptor consisting of the two components CRLR and receptor activator modifying protein 1 (RAMP1) in chondrocytes is scarce [7]. However, previous data from our laboratory demonstrated the expression of CRLR predominantly in the middle and deep zones of articular cartilage chondrocytes from both OA and non-OA patients, suggesting that direct αCGRP signaling could be a potential route of action [33].

Accumulating evidence shows that subchondral bone remodeling contributes directly and indirectly to cartilage destruction and joint pain [34]. However, exercise has been shown to modify the subchondral and metaphyseal trabecular microstructure with the potential to ameliorate OA [13, 35]. According to literature, αCGRP acts as an anabolic factor stimulating osteoblast differentiation, and activity and inhibiting osteoclast activity making it a possible link between joint loading and OA progression [7, 36]. αCGRP KO mice develop osteopenia during growth that is caused by a reduced bone formation rate [37]. Surgical OA-induction via DMM on the other hand results in subchondral bone sclerosis, meniscal ossification and osteophyte formation [3, 13]. Inhibition of αCGRP after DMM surgery was shown to prevent bone sclerosis and cartilage degeneration [38]. We found that DMM surgery did not induce subchondral bone sclerosis 8 weeks after surgery, while αCGRP deficiency led to subchondral and metaphyseal bone sclerosis after moderate exercise. These results are in contradiction to the findings in non-exercise and non-challenged αCGRP KO mice by Schinke et al. and by Nakasa et al. in WT mice after DMM but are fully in line with our own previous work [3, 37, 38]. It should be noted that Schinke et al. analyzed lumbar vertebrae and did not induce OA while Nakasa et al. used a CGRP receptor antagonist (Olcegepant) to inhibit αCGRP signaling after DMM surgery whereas in our set up αCGRP is absent already at birth possibly explaining different outcomes due to different experimental set ups. In addition, we found that intense exercise induced sclerotic changes in the subchondral bone of WT mice but attenuated sclerosis in αCGRP KO mice. In the metaphyseal bone underneath, however, intense exercise led to a significant loss of trabeculae in αCGRP KO mice, while WT mice remained unaffected. These results clearly show that αCGRP plays a crucial role in preventing bone structural changes induced by joint loading and mechanical stress during OA pathogenesis. Consistent with our earlier studies, the diameter of the medial tibial plateau increased significantly in the DMM groups of both genotypes compared to their respective Sham groups. However, no differences were observed between genotypes or exercise intensity levels [3].

OA onset is accompanied by the activation of inflammatory responses of the innate and adaptive immune system. Due to the local inflammation in the joint, the expression of biomarkers in the synovial fluid, serum and urine is altered even before clinical symptoms of OA manifest themselves [39, 40]. We found no differences in proinflammatory and OA-associated serum factors between DMM and Sham mice but a significant increase in serum levels of αCGRP KO mice especially after intense exercise. The lack of DMM-induced differences suggests that this is likely an OA-independent effect only due to the systemic KO of αCGRP. It should be noted, that αCGRP and its receptors are expressed widely throughout the body and αCGRP directly influences macrophages and dendritic cells, reducing their ability to produce inflammatory cytokines and present antigens to T cells in response to injury or infection [41, 42]. Furthermore, αCGRP serum levels are elevated not only in acute pain conditions but also in pain after exercise [9]. This suggests that αCGRP plays a critical role in attenuating inflammatory processes after intense exercise. In αCGRP-KO mice, the lack of αCGRP presumably leads to the increased inflammatory serum levels of the analyzed markers. Although we did not see an effect of the increased proinflammatory serum milieu in αCGRP KO mice on cartilage matrix degradation, it is possible that such an effect may become clear at later time points or is only detectable at the nanoscale (see AFM data).

Surely, it is necessary to point out the limitations of our study and their effect on its outcomes. First, no female animals were included in this study. According to a large body of literature female mice develop less severe OA than male mice after DMM surgery, making them less suitable for PTOA studies [12, 43–45]. However, a recent systematic study found that joint damage of C57Bl/6 mice develops comparably in both females and males after DMM surgery [46]. Given that the prevalence of OA in women is reportedly higher than in men at ages 50 and higher, follow up studies should include female mice in their design [1]. This study also refrained from the use of littermate controls. Instead, αCGRP-/- mice were bred in house, while C57Bl/6J mice were purchased from Charles River for comparison. This introduces the risk of genetic and phenotypic differences between αCGRP-/- and WT mice and increases the chance for false positives [47]. However, this study design follows the required 3R principles by preventing the breeding of mice that carry the wrong phenotype and minimizing unwanted breeding surplus. This study also refrained from including a non-exercise control group, which prevents the investigation of general exercise effects that are already present after moderate exercise. However, using moderate exercise as baseline ensures for all mice a standardized minimum of mechanical load and prevents animals from slacking off due to DMM induced joint instability or pain. Finally, although previous studies have shown that DMM does not significantly alter gait patterns or overall mobility within the observed time frame [8, 48], it cannot be ruled out that treadmill exercise may induce abnormal limb loading in DMM mice. Such changes could influence bone morphology independently of intra-articular pathology. Nonetheless, osteoarthritis and abnormal mechanical loading are two sides of the same coin, making it difficult to fully disentangle their individual contributions.

## Conclusion

In summary, this study highlights the complex interplay between exercise intensity, αCGRP signaling, and PTOA progression. Both, moderate and intense exercise after DMM surgery equally increased cartilage degradation, leading to distinct patterns of ECM stiffening, with αCGRP deficiency preventing these changes. Additionally, αCGRP played a protective role in mitigating exercise-induced bone remodeling, particularly in the subchondral and metaphyseal regions. Elevated inflammatory markers in αCGRP-deficient mice suggest a broader role for αCGRP in modulating systemic responses to mechanical stress induced by running exercise, independent from DMM induced OA. These findings position αCGRP as an intrinsic regulator of joint and bone health during running exercise and OA progression.

## Electronic supplementary material

Below is the link to the electronic supplementary material.

Supplementary Material 1: Supplementary fig.1. Impact of αCGRP deficiency and exercise intensity on cartilage degradation after OA induction. Representative images of Safranin-O stained frontal sections of paraffin embedded knee joints of WT and KO mice exposed to moderate or intense exercise. Cartilage of (A) the medial tibia plateau (MTP) and femoral condyle (MFC) as well as (B) the lateral tibia plateau (LTP) and femoral condyle (LFC) were graded 4 and 8 weeks after Sham or DMM surgery. MM/LM = medial/lateral meniscus.

Supplementary Material 2: Supplementary fig.2. Impact of αCGRP deficiency and exercise intensity on lateral cartilage degradation after OA induction. Cartilage was evaluated for grades of destruction according to the OARSI guidelines for murine OA. Cartilage of the right knee joints of WT and KO mice exposed to moderate or intense exercise were graded 4 weeks (A) and 8 weeks (B) after Sham or DMM surgery. Means of the maximal OARSI scores of the lateral tibial and femoral cartilage were compared. Statistical analysis using Kruskal-Wallis and Dunn’s test for multiple comparisons. * *p* < 0.05, ** *p* < 0.01. *N* = 6–10.

Supplementary Material 3: Supplementary fig.3. Atomic force microscopy-based analysis of the superficial cartilage matrix stiffness in αCGRP deficient mice after OA-induction and forced exercise. Analysis of articular cartilage of the right knee joint of WT and KO mice exposed to moderate and intense exercise at 8 weeks after DMM or Sham surgery. Histograms of Young’s modulus (stiffness) distributions of the superficial zone cartilage matrix. The black line in each histogram represents a fit to the data using a linear combination of two Gaussian distributions. The dashed black lines show the individual Gaussian distributions representing the proteoglycan (left) and the collagen (right) Young’s moduli, respectively, as described in detail in the methods section. *N* = 3.

Supplementary Material 4: Supplementary fig.4. Atomic force microscopy-based analysis of the middle zone cartilage matrix stiffness in αCGRP deficient mice after OA-induction and forced exercise. Analysis of articular cartilage of the right knee joint of WT and KO mice exposed to moderate and intense exercise at 8 weeks after DMM or Sham surgery. Histograms of Young’s modulus (stiffness) distributions of the middle zone cartilage matrix. The black line in each histogram represents a fit to the data using a linear combination of two Gaussian distributions. The dashed black lines show the individual Gaussian distributions representing the proteoglycan (left) and the collagen (right) Young’s moduli, respectively, as described in detail in the methods section. *N* = 3.

Supplementary Material 5: Supplementary fig.5. Effect of αCGRP deficiency and forced exercise on subchondral bone morphology after OA-induction. Ultra-high resolution nanoCT analysis of the subchondral bone of the medial tibia in WT and KO mice exposed to moderate or intense exercise at 8 weeks after DMM or Sham surgery. Analysis of (A) trabecular number (Tb. N.) and (B) subchondral bone plate (SBP) thickness. Statistical analysis using Two-Way ANOVA and Tukey’s multiple comparisons test. * *p* < 0.05, ** *p* < 0.01, *** *p* < 0.001. *N* = 3.

## Data Availability

The datasets used and/or analyzed during the current study are available from the corresponding author on reasonable request.
