# Supplementary figures and images for: Decoding the impact of exercise and αCGRP signaling on murine post-traumatic osteoarthritis progression
Source: Arthritis Res Ther. 2025 Jun 21;27:129. doi: 10.1186/s13075-025-03589-6 (PMC12181913; doi:10.1186/s13075-025-03589-6)

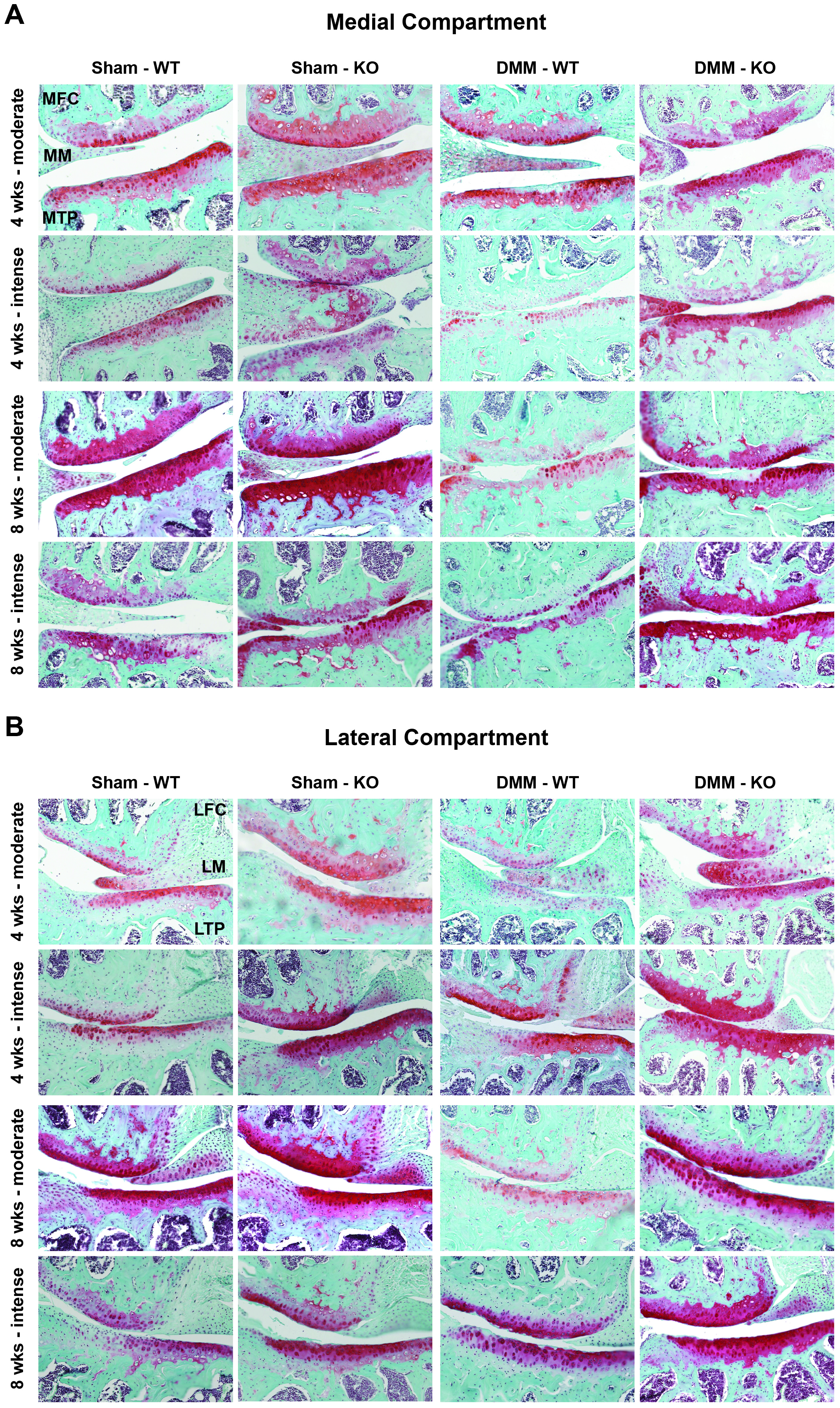

Supplement: Supplementary file 1 — Supplementary Material 1: Supplementary fig.1. Impact of αCGRP deficiency and exercise intensity on cartilage degradation after OA induction. Representative images of Safranin-O stained frontal sections of paraffin embedded knee joints of WT and KO mice exposed to moderate or intense exercise. Cartilage of (A) the medial tibia plateau (MTP) and femoral condyle (MFC) as well as (B) the lateral tibia plateau (LTP) and femoral condyle (LFC) were graded 4 and 8 weeks after Sham or DMM surgery. MM/LM = medial/lateral meniscus. [file 13075_2025_3589_MOESM1_ESM.jpg]

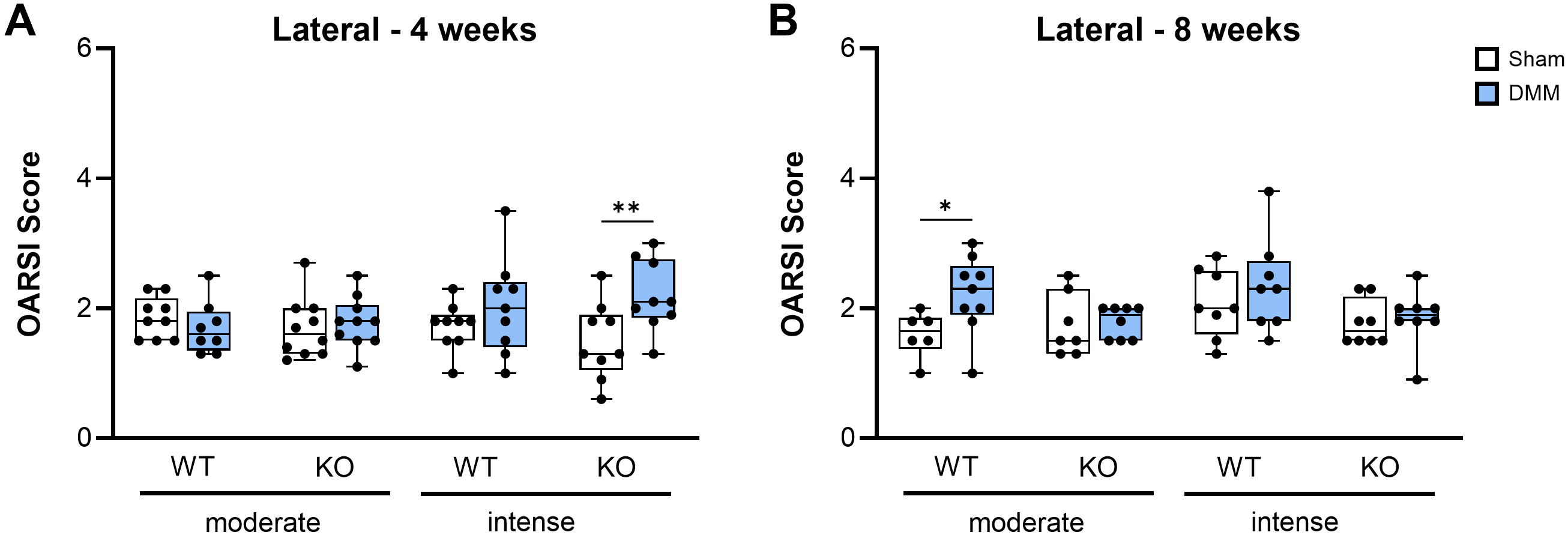

Supplement: Supplementary file 2 — Supplementary Material 2: Supplementary fig.2. Impact of αCGRP deficiency and exercise intensity on lateral cartilage degradation after OA induction. Cartilage was evaluated for grades of destruction according to the OARSI guidelines for murine OA. Cartilage of the right knee joints of WT and KO mice exposed to moderate or intense exercise were graded 4 weeks (A) and 8 weeks (B) after Sham or DMM surgery. Means of the maximal OARSI scores of the lateral tibial and femoral cartilage were compared. Statistical analysis using Kruskal-Wallis and Dunn’s test for multiple comparisons. * p < 0.05, ** p < 0.01. N = 6–10. [file 13075_2025_3589_MOESM2_ESM.png]

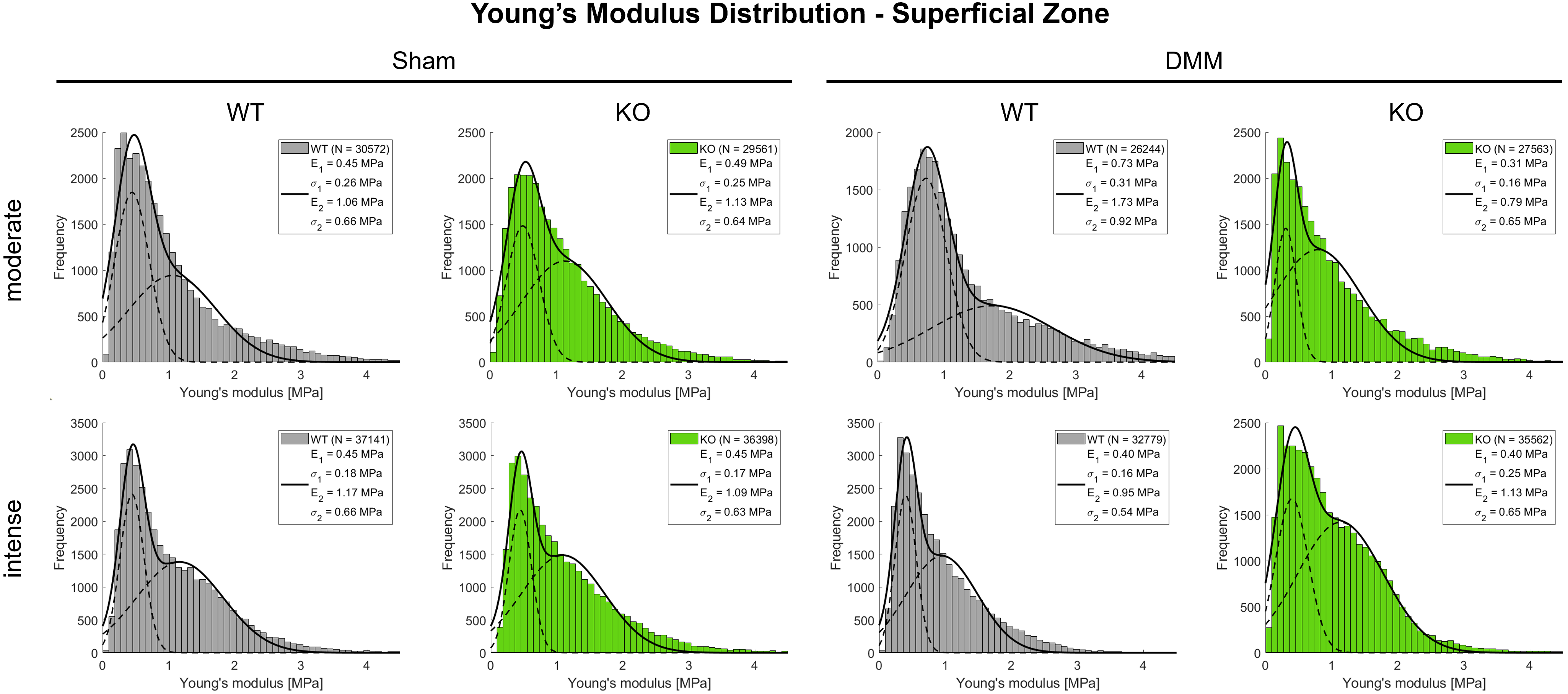

Supplement: Supplementary file 3 — Supplementary Material 3: Supplementary fig.3. Atomic force microscopy-based analysis of the superficial cartilage matrix stiffness in αCGRP deficient mice after OA-induction and forced exercise. Analysis of articular cartilage of the right knee joint of WT and KO mice exposed to moderate and intense exercise at 8 weeks after DMM or Sham surgery. Histograms of Young’s modulus (stiffness) distributions of the superficial zone cartilage matrix. The black line in each histogram represents a fit to the data using a linear combination of two Gaussian distributions. The dashed black lines show the individual Gaussian distributions representing the proteoglycan (left) and the collagen (right) Young’s moduli, respectively, as described in detail in the methods section. N = 3. [file 13075_2025_3589_MOESM3_ESM.png]

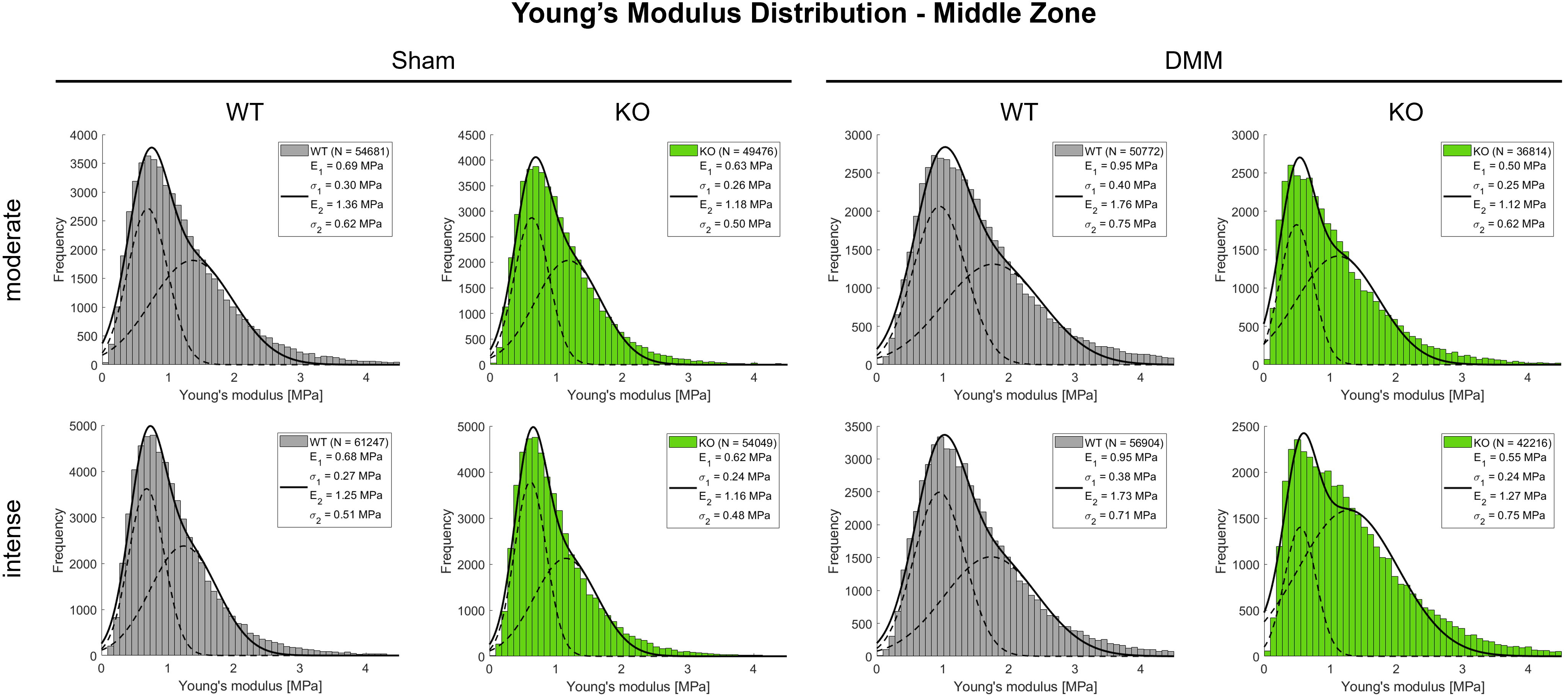

Supplement: Supplementary file 4 — Supplementary Material 4: Supplementary fig.4. Atomic force microscopy-based analysis of the middle zone cartilage matrix stiffness in αCGRP deficient mice after OA-induction and forced exercise. Analysis of articular cartilage of the right knee joint of WT and KO mice exposed to moderate and intense exercise at 8 weeks after DMM or Sham surgery. Histograms of Young’s modulus (stiffness) distributions of the middle zone cartilage matrix. The black line in each histogram represents a fit to the data using a linear combination of two Gaussian distributions. The dashed black lines show the individual Gaussian distributions representing the proteoglycan (left) and the collagen (right) Young’s moduli, respectively, as described in detail in the methods section. N = 3. [file 13075_2025_3589_MOESM4_ESM.png]

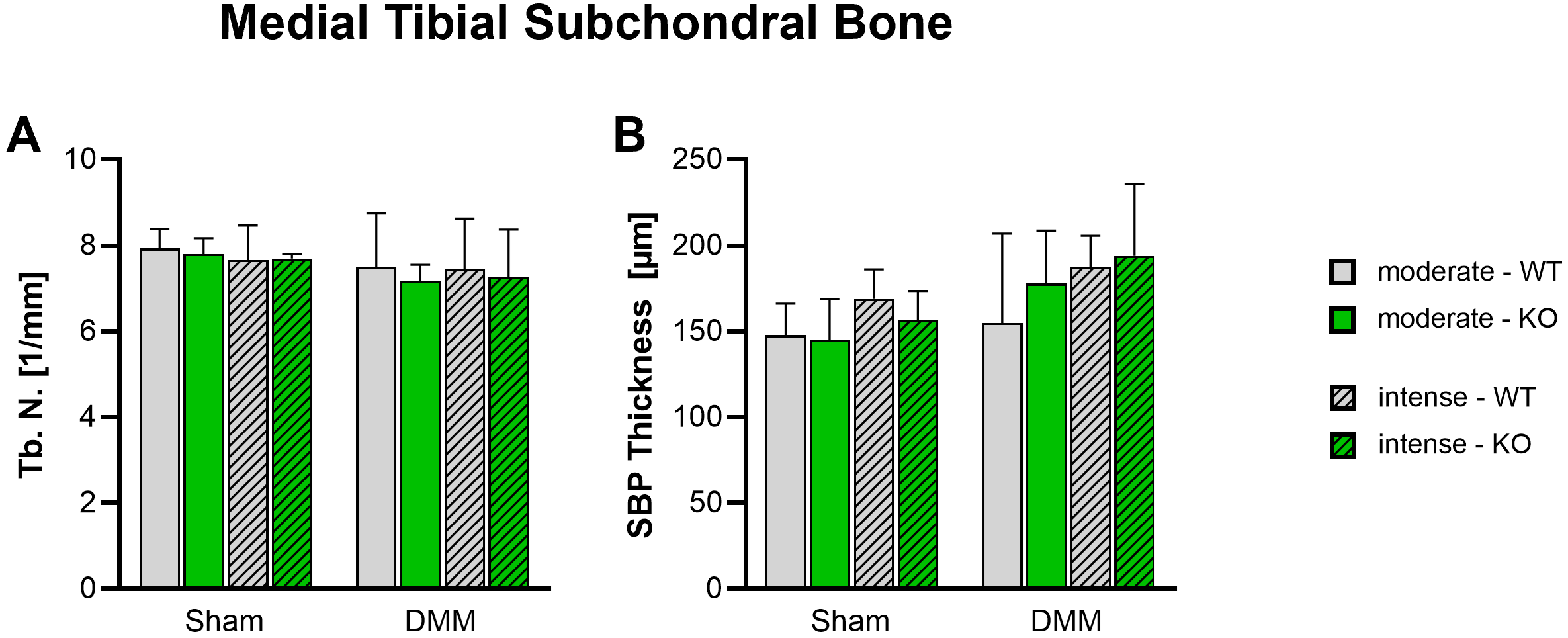

Supplement: Supplementary file 5 — Supplementary Material 5: Supplementary fig.5. Effect of αCGRP deficiency and forced exercise on subchondral bone morphology after OA-induction. Ultra-high resolution nanoCT analysis of the subchondral bone of the medial tibia in WT and KO mice exposed to moderate or intense exercise at 8 weeks after DMM or Sham surgery. Analysis of (A) trabecular number (Tb. N.) and (B) subchondral bone plate (SBP) thickness. Statistical analysis using Two-Way ANOVA and Tukey’s multiple comparisons test. * p < 0.05, ** p < 0.01, *** p < 0.001. N = 3. [file 13075_2025_3589_MOESM5_ESM.png]
